# Effect of Fear of Falling on Mobility Measured During Lab and Daily Activity Assessments in Parkinson’s Disease

**DOI:** 10.3389/fnagi.2021.722830

**Published:** 2021-11-30

**Authors:** Arash Atrsaei, Clint Hansen, Morad Elshehabi, Susanne Solbrig, Daniela Berg, Inga Liepelt-Scarfone, Walter Maetzler, Kamiar Aminian

**Affiliations:** ^1^Laboratory of Movement Analysis and Measurement, Ecole Polytechnique Fédérale de Lausanne (EPFL), Lausanne, Switzerland; ^2^Department of Neurology, UKSH, Christian-Albrechts-University, Kiel, Germany; ^3^Department of Neurodegeneration, Center for Neurology and Hertie-Institute for Clinical Brain Research, University of Tübingen, Tübingen, Germany; ^4^German Center for Neurodegenerative Diseases, Tübingen, Germany; ^5^IB-Hochschule, Stuttgart, Germany

**Keywords:** inertial sensor, wearables, sit-to-stand, gait, turning, timed-up and go

## Abstract

In chronic disorders such as Parkinson’s disease (PD), fear of falling (FOF) is associated with falls and reduced quality of life. With inertial measurement units (IMUs) and dedicated algorithms, different aspects of mobility can be obtained during supervised tests in the lab and also during daily activities. To our best knowledge, the effect of FOF on mobility has not been investigated in both of these settings simultaneously. Our goal was to evaluate the effect of FOF on the mobility of 26 patients with PD during clinical assessments and 14 days of daily activity monitoring. Parameters related to gait, sit-to-stand transitions, and turns were extracted from IMU signals on the lower back. Fear of falling was assessed using the Falls Efficacy Scale-International (FES-I) and the patients were grouped as with (PD-FOF+) and without FOF (PD-FOF−). Mobility parameters between groups were compared using logistic regression as well as the effect size values obtained using the Wilcoxon rank-sum test. The peak angular velocity of the turn-to-sit transition of the timed-up-and-go (TUG) test had the highest discriminative power between PD-FOF+ and PD-FOF− (*r*-value of effect size = 0.61). Moreover, PD-FOF+ had a tendency toward lower gait speed at home and a lower amount of walking bouts, especially for shorter walking bouts. The combination of lab and daily activity parameters reached a higher discriminative power [area under the curve (AUC) = 0.75] than each setting alone (AUC = 0.68 in the lab, AUC = 0.54 at home). Comparing the gait speed between the two assessments, the PD-FOF+ showed higher gait speeds in the capacity area compared with their TUG test in the lab. The mobility parameters extracted from both lab and home-based assessments contribute to the detection of FOF in PD. This study adds further evidence to the usefulness of mobility assessments that include different environments and assessment strategies. Although this study was limited in the sample size, it still provides a helpful method to consider the daily activity measurement of the patients with PD into clinical evaluation. The obtained results can help the clinicians with a more accurate prevention and treatment strategy.

## Introduction

Parkinson’s disease (PD) is a neurodegenerative disease that is associated with the degeneration of the dopaminergic nerve cells in the substantia nigra (Rai and Singh, 2020). Although currently, there is no cure for PD, treatment options such as Levodopa focus on alleviating PD symptoms. Fear of falling (FOF) is one of the most stressful symptoms for patients with PD (Frazier, 2000; Jonasson et al., 2018), leading to reduced quality of life and social isolation (Howcroft et al., 2013). Moreover, it is the strongest predictor currently known for future falls in this population (Lindholm et al., 2015), which indirectly but strongly associates FOF with the consequence of falls, such as fractures and other injuries (Bloem et al., 2001; Allen et al., 2013).

Fear of falling can be assessed by several scales of which the Falls Efficacy Scale-International (FES-I) is the most widely used to evaluate the concerns of patients about falling during various daily activities (Delbaere et al., 2010). These activities include walking, postural transitions, and turnings during daily activities. Being subjective in nature, FOF can have impacts on mobility that can be measured objectively (Rochat et al., 2010). Therefore, by the assessment of mobility, future falls can be predicted (Delbaere et al., 2004). Inertial measurement units (IMUs) enable the objective evaluation of mobility performance, both during functional tests in the lab and during daily activities. Instrumenting functional tests such as the timed-up-and-go (TUG) and five-time sit-to-stand (5xSTS) with IMUs provide a more in-depth analysis of gait and balance performance (Salarian et al., 2010; Van Lummel et al., 2016). Furthermore, IMUs can also help clinicians to evaluate the performance of patients during daily activities that are often very different from the supervised assessment in the lab and the clinic (Warmerdam et al., 2020).

The potential of IMUs to distinguish patients with falls from those without has already been shown (Howcroft et al., 2013). These studies suggest that the most promising mobility parameters to detect an increased risk of falling are in the area of gait (Marschollek et al., 2009; Greene et al., 2010; Weiss et al., 2011, 2013), postural transition (Najafi et al., 2002; Narayanan et al., 2008; Doheny et al., 2011; Weiss et al., 2011), and turning (Haertner et al., 2018). However, none of these studies investigated the contribution of FOF to these associations in detail.

In community-dwelling older adults, it has been shown that IMU-derived TUG parameters, such as total duration, turning velocity, and sit-to-stand duration, have a significant association with the FES-I total score (Williams and Nyman, 2018). Moreover, it has been shown in patients with PD that FOF affects their turning performance during the TUG test (Haertner et al., 2018). Patients with PD with FOF had significantly lower turning peak angular velocity, and PD fallers had significantly lower gait speed, compared to non-fallers (Latt et al., 2009). A drawback of the previous studies is that the performance of the participants has been studied mostly during assessments performed in the clinic while the association between FOF and the performance of the investigated cohorts during daily activities remains unknown. This is an enormous disadvantage, as daily activity and mobility are influenced by psychological and environmental factors that cannot be effectively investigated in a supervised environment (Owsley and McGwin, 2004; Feltz and Payment, 2005; Rudman et al., 2006; Kaspar et al., 2015; Evers et al., 2020; Shah et al., 2020b; Del Din et al., 2021; Maetzler et al., 2021).

Based on these findings, the first goal of this study was to determine whether there exist mobility differences between patients with PD with (PD-FOF+) and without FOF (PD-FOF−). For this purpose, we compared the IMU-derived gait, sit-to-stand, and turning parameters from the respective lab and daily activity assessments. The second goal was to determine whether daily activity assessment can complement lab assessment in differentiating PD-FOF+ from PD-FOF−. The third goal was to investigate the associations between the same parameters obtained during these two assessment settings and study their differences in PD-FOF+ and PD-FOF−.

## Materials and Methods

### Participants and Study Cohort

Twenty-six participants with PD were included in the analysis. The inclusion criteria were age between 50 and 85 years, PD based on the United Kingdom Brain-Bank Society criteria, and the ability to understand and communicate well with the investigator. Patients with dementia were excluded from the study (Emre et al., 2007). All participants gave their written informed consent and the study was approved by the ethics committee of the Medical Faculty of the University of Tübingen (protocol no. 686/2013BO1) (Haertner et al., 2018).

### Lab Assessments

Lab assessments were performed during ON medication state and included the Unified Parkinson’s Disease Rating Scale (UPDRS-III) (Goetz et al., 2008) and the Hoehn and Yahr (H&Y) score (Hoehn and Yahr, 2001). Fear of falling was assessed with the FES-I (Yardley et al., 2005). An FES-I score > 19 was defined as the presence of FOF (Delbaere et al., 2010). The patients were also evaluated for depressive symptoms (Beck’s Depression Inventory, BDI), the amount of Levodopa equivalent dose, and quality of life (Parkinson’s Disease Questionnaire, PDQ-39).

For the mobility assessments, the participants were equipped with Mobility Lab^®^ (APDM, Portland, United States) IMUs on the lower back and the two feet. The sampling frequency was set at 128 Hz. For the analysis, accelerometer and gyroscope data were used. All participants performed first a 7-m TUG test at their convenient speed. The TUG test includes a sit-to-stand movement, a walking phase, a 180° turn, a walking back phase, and a turn-to-sit movement. The turn-to-sit transition consists of a simultaneously performed stand-to-sit transition and a 180° turn. Then, the participants performed the 5xSTS test once with their preferred speed and once as fast as possible. Rest periods were given between these three lab mobility tests.

For the analysis of the TUG test, the lower back IMU was used to analyze the sit-to-stand and stand-to-sit postural transitions with a previously validated algorithm (Atrsaei et al., 2020). The beginning of the sit-to-stand (*t*_*b,SiSt*_) and the end of the stand-to-sit (*t*_*e,StSi*_) times, as well as the sit-to-stand peak power (*P*_*TUG*_) were extracted. The two turns within the TUG were analyzed by another validated algorithm, using the data from the lower back IMU (Salarian et al., 2010). The end of the second turn (*t*_*e,Turn2*_), as well as the maximum angular velocities around the vertical axis of each of the two turns (ω_*TUG*,1_ and ω_*TUG*,2_) were extracted. The total time of the TUG was calculated by subtracting the start of the sit-to-stand from the maximum value between the end of the second turn and the end of stand-to-sit: *T*_*TUG*_ = Max(*t*_*e*,*StSi*_,*t*_*e*,*Turn*2_)−*t*_*b*,*SiSt*_.

The IMUs on the lower back and feet were used to extract the instantaneous gait speed during the TUG test based on the algorithm introduced in Atrsaei et al. (2021b). The mean gait speed of the whole test was calculated (*V*_*TUG*,*avg*_).

The 5xSTS tests were analyzed by the algorithm given in Atrsaei et al. (2020), using the data obtained from the lower back IMU. The following parameters were calculated: total time and mean sit-to-stand peak power of the normal (*T*_*5xSTS,N*_, *P*_*5xSTS,N*_) and the fast 5xSTS (*T*_5*xSTS*,*F*_,*P*_5*xSTS*,*F*_).

### Mobility Assessment During Daily Activities

The participants were equipped with a RehaGait^®^ IMU (Hasomed, Magdeburg, Germany) in an elastic belt on the lower back and were asked to wear the system for 14 days. The patients were instructed to plug the sensor into a personal computer to charge during the night. The following morning, the patients were asked to unplug the sensor and wear it. The data recording was started automatically right after the sensor is unplugged. Measurement phases of less than 6 h/day were discarded from the analysis. The following mobility parameters were extracted for each patient.

#### Gait

Walking bouts were detected by the algorithm introduced in Atrsaei et al. (2021b). Instantaneous gait speed, i.e., gait speed at each second was calculated (Atrsaei et al., 2021b). Instances in which the gait speed was less than 0.2 m/s were not included in the walking bouts as these instances can be considered as “non-gait” periods (Atrsaei et al., 2021a). Walking bouts of less than 15 s were excluded from the analysis, to have a more steady-state gait and prevent non-locomotion movements to be detected (Atrsaei et al., 2021a). The total duration of walking for each day was obtained and was expressed as the percentage of the measurement duration of the respective day. Over all the days of measurement, the minimum (*Gait*_*ALL,min*_), average (*Gait*_*ALL,avg*_), and maximum (*Gait*_*ALL,max*_) values of the walking percent were calculated. For instance, when a participant was assessed over a period of 5 days, and walked 5, 10, 15, 20, and 25% of the entire daily assessment periods, respectively, the *Gait*_*ALL,min*_, *Gait*_*ALL,avg*_, and *Gait*_*ALL,max*_ would be 5, 15, and 25%, respectively.

The walking bouts were divided into short (between 15 and 30 s), medium (between 30 and 60 s), and long ones (longer than 60 s). Again, the minimum, average, and maximum values of the walking percentage per day for each type of walking bout were calculated. The indices *SWB*, *MWB*, and *LWB* were used to describe short, medium, and long walking bouts.

Over all the days of measurement stacked together, the gait speed distribution during all the walking bouts (*V*_*ALL*_), as well as during the short (*V*_*SWB*_), medium (*V*_*MWB*_), and long (*V*_*LWB*_) walking bouts were obtained separately. For each of these four distributions, the median, and the 95th percentile values were calculated.

There is evidence in the literature that gait speed often has a bimodal distribution during daily activities (Van Ancum et al., 2019; Atrsaei et al., 2021a). The first mode represents the lower preferred gait speed of the participants while the second mode represents the higher preferred gait speed of the participants (Van Ancum et al., 2019). Therefore, we also extracted the first and second modes of *V*_*ALL*_ distribution as *V*_*μ _1*_ and *V*_*μ _2*_, respectively.

#### Sit-to-Stand Transitions

Sit-to-stand transitions were detected during daily activities with a validated algorithm (Atrsaei et al., 2020). For each day, the number of sit-to-stands per hour was obtained. The minimum (*SiSt*_*min*_), average (*SiSt*_*avg*_), and maximum (*SiSt*_*max*_) number of sit-to-stands per hour were calculated over all days of measurement. Furthermore, for each sit-to-stand, the vertical peak power was determined as this parameter is a predictor of prospective falls (Regterschot et al., 2014). The distribution of all the peak power values over all the days of measurement stacked together was obtained as *P*_*H*_. The median of this distribution (*P*_*H,P50*_) and its 95th percentile (*P*_*H,P95*_) were calculated.

#### Turns

Turns were detected during daily activities with a validated algorithm (El-Gohary et al., 2014). The number of turns per hour was determined for each day. The minimum (*Turns*_*min*_), average (*Turns*_*avg*_), and maximum (*Turns*_*max*_) number of turns per hour was also calculated over all the days of measurement. For each turn, the peak angular velocity around the vertical direction was obtained. The distribution of all the peak angular velocity values over all the days of measurement stacked together were obtained as ω_*H*_. The median (ω_*H*,*P*50_) and 95th percentile (ω_*H*,*P*95_) of this distribution were calculated.

### Comparison Between PD-FOF+ and PD-FOF−

All the mobility parameters extracted from the lab and daily activity assessments were compared between PD-FOF+ and PD-FOF−. To exclude the potential differences due to gender and PD stage, the values were adjusted for gender and UPDRS-III with a multivariable logistic regression model. This analysis determines the odds of being PD-FOF+ considering gender (binary value, 0 for male, 1 for female), UPDRS-III (real-valued), and one of the mobility parameters (real-valued) explained in the previous section as independent variables. Moreover, the effect size (ES) (obtained by the *r*-value) was obtained by dividing the Wilcoxon rank-sum test statistics by the square root of the population (Ivarsson et al., 2013). An *r* value of about 0.1 indicates a small, 0.3 a medium, and 0.5 a large effect size, respectively (Cohen, 1992).

### Fear of Falling Classification

To determine the predictive power of the extracted parameters in classifying PD-FOF+ and PD- FOF−, three classifiers based on a decision tree were used. Each classifier was trained based on one of the three sets of features mentioned below:

• *𝔽*1, Lab and daily activity (selected features): From all the parameters extracted from the lab and daily activity measurements, we selected those with an absolute *r* value of higher than 0.2. A backward elimination method was further applied to select the optimal features (Dadashi et al., 2014).
• *𝔽*2, Lab: From the set *𝔽*1, the parameters from the lab assessment were used.
• *𝔽*3, Daily activity: From the set *𝔽*1, the parameters from daily activity assessment were used.

The decision tree approach was used due to its proven performance in classifying patient populations based on mobility biomarkers (Millor et al., 2017; Rehman et al., 2019). For all the three sets mentioned above, cross-validation was performed based on the leave-one subject-out approach. The classification performance was evaluated by sensitivity, specificity, precision, accuracy, and area under the receiver operating characteristic curve (AUC) metrics.

### Lab Versus Daily Activity Assessment

For each of the two groups, the gait speed, sit-to-stand peak power, and peak angular velocity were compared between lab and daily activities. For each parameter, a paired comparison was performed with the Wilcoxon sign rank test, and the significance level was set at *p* = 0.05. Pearson’s correlation coefficient (ρ) was also obtained. A correlation coefficient < 0.5 was considered as low, between 0.5 and 0.7 as moderate, and >0.7 as high (Mukaka, 2012).

Moreover, each parameter obtained during the daily activities was divided by the same parameter obtained during the lab assessment. The new unitless parameters were compared between PD-FOF+ and PD-FOF− by the Wilcoxon rank-sum test.

## Results

### Comparison Between PD-FOF+ and PD-FOF−

The characteristics of the participants are shown in Table 1. Of the 26 participants, nine had an FES-I score > 19. The PD-FOF+ showed a trend toward higher UPDRS-III scores in comparison with the PD-FOF−. The Levodopa equivalent dose, as well as the BDI score, was not significantly different between the two groups. However, the PDQ scale, as well as its mobility subpart, were significantly different between PD-FOF+ and PD-FOF− (*p* < 0.001 and = 0.0014, respectively).

**TABLE 1:** Comparison of PD-FOF+ and PD-FOF−.

**Parameter**	**PD-FOF+**	**PD-FOF−**	***p*-value**	**ES**
Number	9 (9 females)	17 (12 females)	0.083	0.35
Age (year)	65 [62, 69]	64 [58, 75]	0.829	0.05
Height (m)	1.78 [1.67, 1.83]	1.75 [1.69, 1.79]	0.608	0.11
Weight (kg)	81.0 [77.0, 86.0]	77 [70.5, 97.0]	0.935	−0.02
UPDRS-III (0-132)	30 [24, 34]	22 [18, 28]	0.053	0.38

*The *p*-value was obtained by Wilcoxon rank sum test. Significance level was set at 0.05. Except the number of participants, the values are shown with Median [IQR]. ES is the effect size obtained by the *r*-value.*

Table 2 presents results from the comparison of the lab and daily activity mobility parameters between PD-FOF+ and PD-FOF−. After the adjustment for UPDRS-III and gender, PD-FOF+ participants had significantly longer *T*_*TUG*_ accompanied by slower ω_*TUG*,1_, ω_*TUG*,2_, and a slower *V*_*TUG,avg*_ which, however, did not reach significance.

**TABLE 2:** Comparison of the extracted parameter between the group with fear of falling (FOF) (PD-FOF+) and without (PD-FOF−).

**Category**	**Parameter**	**PD-FOF+**	**PD-FOF−**	***p*-value**	**ES**
TUG	*T*_*TUG*_ (s)	19.93 [19.29, 21.43]	17.40 [15.23, 19.38]	0.044*	0.51
	*V*_*TUG,avg*_ (m/s)	1.01 [0.95, 1.08]	1.13 [1.05, 1.29]	0.069	−0.47
	ω_*TUG*,1_ (deg/s)	124.4 [119.1, 165.0]	161.6 [149.4, 202.0]	0.029*	−0.36
	ω_*TUG*,2_ (deg/s)	110.2 [103.0, 132.4]	158.5 [140.2, 167.3]	0.018*	−0.61
	*P*_*TUG*_ (W)	44.11 [16.07, 49.26]	37.32 [28.61, 45.08]	0.269	−0.01
Normal 5xSTS	*T*_*5xSTS,N*_ (s)	17.02 [15.79, 21.61]	16.94 [15.05, 21.11]	0.772	−0.10
	*P*_*5xSTS,N*_ (W)	44.86 [32.96, 51.29]	39.13 [32.47, 50.02]	0.949	0.33
Fast 5xSTS	*T*_*5xSTS,F*_ (s)	14.24 [13.58, 15.02]	11.48 [11.03, 16.32]	0.594	0.35
	*P*_*5xSTS,F*_ (W)	65.32 [46.86, 74.92]	49.16 [37.71, 70.15]	0.373	0.35
Gait at Home	*V*_*ALL,P50*_ (m/s)	0.81 [0.79, 0.93]	0.88 [0.76, 0.93]	0.660	0.01
	*V*_*ALL,P95*_ (m/s)	1.17 [1.11, 1.33]	1.23 [1.09, 1.27]	0.822	0.13
	*V*_*μ _1*_ (m/s)	0.49 [0.36, 0.59]	0.63 [0.43, 0.75]	0.053	−0.31
	*V*_*μ _2*_(m/s)	0.91 [0.82, 1.00]	0.96 [0.84, 1.06]	0.447	−0.16
	*Gait*_*ALL,min*_ (%)	0.47 [0.43, 0.83]	0.93 [0.62, 2.86]	0.122	−0.29
	*Gait*_*ALL,avg*_ (%)	3.10 [2.69, 3.33]	4.28 [2.78, 5.95]	0.174	−0.24
	*Gait*_*ALL,max*_ (%)	6.59 [4.56, 7.88]	8.05 [6.71, 13.34]	0.177	−0.33
Sit-to-stand at Home	*P*_*H,P50*_ (W)	18.72 [12.62, 24.33]	19.54 [13.00, 25.72]	0.291	−0.05
	*P*_*H,P95*_ (W)	43.10 [36.69, 54.54]	43.22 [33.30, 62.99]	0.239	−0.03
	*SiSt*_*min*_ (/h)	1.75 [0.96, 2.60]	1.75 [0.80, 3.27]	0.998	−0.05
	*SiSt*_*avg*_ (/h)	3.74 [2.80, 4.60]	4.45 [3.42, 5.17]	0.670	−0.24
	*SiSt*_*max*_ (/h)	5.42 [4.11, 6.39]	6.27 [5.59, 8.40]	0.254	−0.33
Turn at Home	ω_*H*,*P*50_ (deg/s)	60.24 [58.67, 63.78]	63.55 [59.13, 68.70]	0.638	−0.23
	ω_*H*,*P*95_ (deg/s)	110.6 [107.2, 123.1]	111.1 [108.1, 123.4]	0.758	−0.03
	*Turns*_*min*_ (/h)	55.71 [48.56, 68.32]	74.30 [55.86, 83.79]	0.923	−0.33
	*Turns*_*avg*_ (/h)	86.46 [82.02, 96.16]	102.5 [85.81, 118.2]	0.578	−0.24
	*Turns*_*max*_ (/h)	123.7 [109.4, 192.1]	142.0 [124.4, 163.0]	0.553	0.10

The *p*-value shows the significance of the coefficient of the inertial measurement unit (IMU)-based parameter in the logistic regression. *P < 0.05 was considered significant. The values of IMU-based parameters are shown by Median [IQR]. The effect size obtained by the *r*-value is ES.

Several parameters were slightly different between the two populations although the logistic regressions showed no statistical significance. For instance, compared with PD- FOF−, PD-FOF+ had, on average, lower gait speeds during the TUG (*V*_*TUG,avg*_) and daily activities (*V*_*μ _1*_), longer *T*_*5xSTS,F*_, lower percentages of walking bouts (i.e., *Gait*_*ALL,min*_, *Gait*_*ALL,avg*_, and *Gait*_*ALL,max*_), and lower numbers of sit-to-stands (*SiSt*_*max*_) and turns (*Turns*_*min*_) per hour during daily activities.

No significant differences were found between the two groups when dividing the walking bouts based on their duration (Table 3). However, PD-FOF+ tended to have a lower percentage of short (e.g., *Gait*_*SWB,max*_) and long (e.g., *Gait*_*LWB,max*_) walking bouts, compared with PD-FOF− (Table 3).

**TABLE 3:** Comparison of the extracted parameter for short, medium, and long walking bouts (WB) between PD-FOF+ and PD-FOF−.

**WB**	**Parameter**	**PD-FOF+**	**PD-FOF−**	***p*-value**	**ES**
Short	*V*_*SWB,P50*_ (m/s)	0.70 [0.63, 0.71]	0.71 [0.60, 0.76]	0.533	0.04
	*V*_*SWB,P95*_ (m/s)	1.10 [1.06, 1.19]	1.07 [1.01, 1.20]	0.529	0.10
	*Gait*_*SWB,min*_ (%)	0.43 [0.39, 0.49]	0.69 [0.36, 1.31]	0.187	−0.24
	*Gait*_*SWB,avg*_ (%)	1.40 [1.19, 1.68]	1.72 [1.30, 2.47]	0.167	−0.26
	*Gait*_*SWB,max*_ (%)	2.65 [2.19, 2.81]	3.15 [2.31, 4.50]	0.095	−0.23
Medium	*V*_*MWB,P50*_ (m/s)	0.83 [0.79, 0.89]	0.85 [0.78, 0.92]	0.761	0.09
	*V*_*MWB,P95*_ (m/s)	1.12 [1.08, 1.27]	1.16 [1.06, 1.24]	0.613	0.12
	*Gait*_*MWB,min*_ (%)	0.00 [0.00, 0.07]	0.00 [0.00, 0.13]	0.271	−0.19
	*Gait*_*MWB,avg*_ (%)	0.51 [0.29, 0.67]	0.62 [0.52, 0.92]	0.110	−0.25
	*Gait*_*MWB,max*_ (%)	1.38 [0.84, 1.88]	1.57 [1.24, 2.35]	0.184	−0.22
Long	*V*_*LWB,P50*_ (m/s)	0.92 [0.89, 1.05]	0.96 [0.89, 1.07]	0.859	0.03
	*V*_*LWB,P95*_ (m/s)	1.24 [1.12, 1.40]	1.24 [1.11, 1.32]	0.772	0.15
	*Gait*_*LWB,min*_ (%)	0.00 [0.00, 0.00]	0.00 [0.00, 0.05]	0.279	−0.16
	*Gait*_*LWB,avg*_ (%)	1.08 [0.74, 1.24]	1.87 [0.78, 2.70]	0.431	−0.28
	*Gait*_*LWB,max*_ (%)	3.24 [2.26, 5.31]	5.43 [3.94, 7.08]	0.314	−0.31

The *p*-value shows the significance of the coefficient of the IMU-based parameter in the logistic regression. *P < 0.05 was considered significant. The values of IMU-based parameters are shown by Median [IQR]. The effect size obtained by the *r*-value is ES.

**FIGURE 1:**
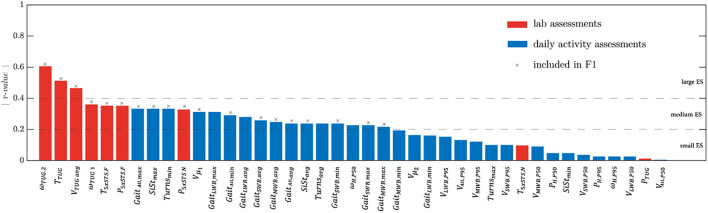
Absolute effect size values (*r*-value) of the mobility parameters extracted from the lab (in red) and daily activity assessments (in blue). The features selected for the *𝔽*1 feature set are marked by a cross (see section “Fear of Falling Classification”).

The effect sizes of the parameters are shown in Table 2 and Figure 1 in descending order. As a general note, lab-extracted parameters showed higher effect sizes than those extracted from the daily activity assessment. ω_*TUG*,2_ had the highest effect size, followed by other parameters extracted from the TUG test (except *P*_*TUG*_ which had a very small effect size, see also Figure 1). ω_*TUG*,1_ had a lower effect size than ω_*TUG*,2_. Directly after the TUG test, parameters ranked the *T*_*5xSTS,F*_ and *P*_*5xSTS,F*_ from the 5xSTS test with fast speed. The effect sizes of the parameters from the 5xSTS with normal speed (*T*_*5xSTS,N*_ and *P*_*5xSTS,N*_) were lower than those from the fast version. *T*_*5xSTS,N*_ had a smaller effect size compared with *P*_*5xSTS,N*_. *Gait*_*ALL,max*_, *SiSt*_*max*_, *Turns*_*min*_, and *V*_*μ _1*_ had the highest effect sizes among the daily activity parameters, and the median gait speed (*V*_*ALL*,*P*50_) the lowest.

### Fear of Falling Classification

Out of the 41 mobility parameters, 23 had an effect size > 0.2 (Figure 1). From these parameters, 19 features (used for machine learning-based classifier; marked with ^*x*^ in Figure 1) were selected by the backward elimination method and used for the *𝔽*1 set.

Based on the three sets of features mentioned in the “Fear of Falling Classification” section, the results of the classification are shown in Table 4. The best performance was achieved based on set *𝔽*1 which was a combination of features obtained from the lab and daily activity assessments (AUC = 0.75). The accuracy of this set was higher than when using lab (*𝔽*2, AUC = 0.68) or daily activity (*𝔽*3, AUC = 0.54) features alone.

**TABLE 4:** The performance metrics of the classification of PD-FOF+ vs. PD-FOF−.

**Feature set**	**Sensitivity (%)**	**Specificity (%)**	**Precision (%)**	**Accuracy (%)**	**AUC**
*𝔽*1, Lab and daily activity	55.6	94.1	83.3	80.8	0.75
*𝔽*2, Lab	57.7	64.7	40.0	65.4	0.68
*𝔽*3, Daily activity	44.4	76.5	50.0	57.7	0.54

*𝔽1: Selected 19 features marked with crosses in Figure 1. *𝔽*2: 7 lab features from *𝔽*1. *𝔽*3: 12 daily activity features from *𝔽*1.*

The sensitivity of the classification based on the features from the lab (*𝔽*2) was higher than that obtained from the daily activity features, while the specificity of the classification based on daily activity features (*𝔽*3) was higher. Moreover, *𝔽*2 features achieved higher accuracy and AUC values, than the *𝔽*3 features.

### Lab Versus Daily Activity Assessment

The results of the paired comparison between lab and daily activity assessments for gait speed, sit-to-stand peak power, and turning peak angular velocity are shown in Table 5. In the PD-FOF+ group, no significant correlations were found between lab and daily activity assessments concerning gait speed. Moreover, PD-FOF+ had significantly higher gait speeds at the 95th percentile of their walking speed distributions compared with the lab (*V*_*ALL,P95*_, *V*_*SWB,P95*_, *V*_*MWB,P95*_, and *V*_*LWB,P95*_). In the PD-FOF− group, *V*_*TUG,avg*_ had a significant but low correlation with *V*_*H,P95*_ (ρ = 0.48). A high correlation was also observed between *V*_*TUG,avg*_ and *V*_*μ _2*_ (ρ = 0.70). Moderate correlations were observed between *V*_*TUG,avg*_ and a gait speed of medium (*V*_*MWB,P95*_) and long (*V*_*LWB,P50*_) walking bouts (ρ = 0.59 and ρ = 0.57, respectively). The PD-FOF− group walked significantly faster during the TUG than during their daily activities.

**TABLE 5:** Paired comparison of the parameters between lab and home.

**Category**	**Lab**	**Daily activity**	**Difference, *p*-value**		**Correlation (ρ)**	
			**PD-FOF+**	**PD-FOF**−	**PD-FOF+**	**PD-FOF**−
Gait speed	*V*_*TUG,avg*_	*V*_*ALL,P50*_	0.023*	< 0.001*	–0.34	0.36
		*V*_*ALL,P95*_	0.008*	0.836	–0.15	0.48*
		*V*_*μ _1*_	0.008*	< 0.001*	0.35	0.44
		*V*_*μ _2*_	0.039*	< 0.001*	–0.03	0.70*
		*V*_*SWB,P50*_	0.008*	< 0.001*	–0.14	0.38
		*V*_*SWB,P95*_	0.039*	0.044*	0.20	0.40
		*V*_*MWB,P50*_	0.016*	< 0.001*	–0.48	0.44
		*V*_*MWB,P95*_	0.008*	0.309	–0.04	0.59*
		*V*_*LWB,P50*_	0.148	< 0.001*	–0.12	0.57*
		*V*_*LWB,P95*_	0.008*	0.193	–0.10	0.42
Sit-to-stand peak power	*P*_*TUG*_	*P*_*H,P50*_	0.039*	0.001*	0.75*	0.48
		*P*_*H,P95*_	0.541	0.006*	0.77*	0.79*
	*P*_*5xSTS,N*_	*P*_*H,P50*_	0.015*	0.003*	0.83*	0.13
		*P*_*H,P95*_	0.578	0.167	0.83*	0.70*
	*P*_*5xSTS,F*_	*P*_*H,P50*_	0.031*	0.002*	–0.28	0.12
		*P*_*H,P95*_	0.312	0.492	–0.34	0.61
Turning peak angular velocity	ω_*TUG,1*_	ω_*H*,*P*50_	< 0.001*	< 0.001*	0.28	0.20
		ω_*H*,*P*95_	0.139	< 0.001*	–0.44	0.00
	ω_*TUG,2*_	ω_*H*,*P*50_	< 0.001*	< 0.001*	0.69	0.43
		ω_*H*,*P*95_	0.815	< 0.001*	0.57	0.00

p-value from the Wilcoxon sign rank test and Pearson’s correlation coefficient (ρ) describe the differences of the parameters between lab and daily life. The significance level was set to 0.05 and shown with *. Significant correlation coefficients were marked with *.

Regarding the sit-to-stand peak power, a high and significant correlation was found between *P*_*TUG*_ and *P*_*H,P95*_ for both groups (PD-FOF+, ρ = 0.77; PD- FOF−, ρ = 0.79). In both groups, *P*_*5xSTS,N*_ had a high and significant correlation with *P*_*H,P95*_ (PD-FOF+, ρ = 0.83; PD- FOF−, ρ = 0.70). No significant correlations were found between the 5xSTS with fast speed and daily activity assessment. Both groups had significantly higher peak power during the 5xSTS tests compared with *P*_*H,P50*_ during daily activities. However, *P*_*H,P95*_ values were not significantly different from the 5xSTS tests in the lab.

Finally, for turning peak angular velocity, no significant correlations were found between the lab and daily activities in any group. For PD-FOF+, there were no significant differences between ω_*H*,*P*95_ and both turns of the TUG. However, PD-FOF− had faster turns in the lab, compared with the home environment.

**FIGURE 2:**
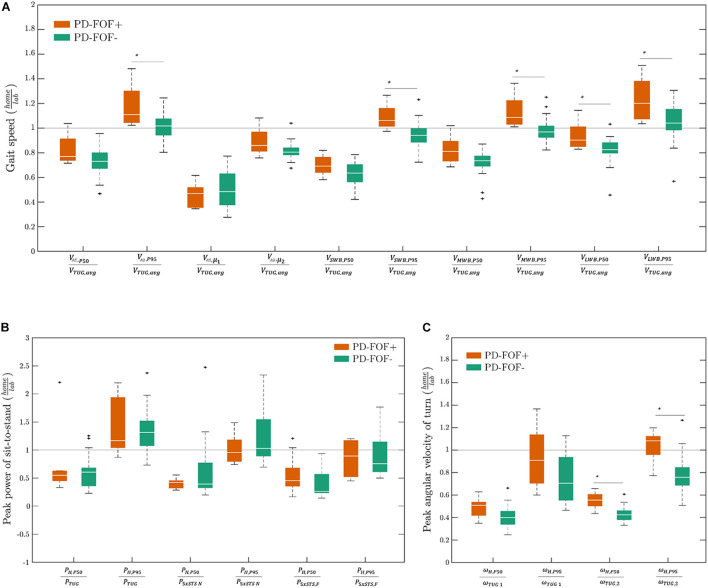
Unitless daily activity divided by lab parameter ratios of **(A)** gait speed, **(B)** sit-to-stand peak power, and **(C)** turning peak angular velocity in PD-FOF+ and PD-FOF−. Differences between the groups were analyzed by the Wilcoxon rank-sum test. Only significant differences were shown on the plots ^∗^*p* < 0.05.

For a better representation of lab versus daily activity parameters, the gait speed, sit-to-stand peak power, and turning peak angular velocity are presented in Figure 2 as unitless ratios (daily activity parameter divided by the respective lab parameter). Most of the ratios were less than 1 (i.e., lower value of a parameter in the daily life environment). However, a few parameters, e.g., $\frac{V_{A\,L\,L,P\,95}}{V_{T\,U\,G,a\,v\,g}}$, $\frac{P_{A\,L\,L,P\,95}}{P_{T\,U\,G}},$ had values > 1, preferentially in the PD-FOF+ group. Moreover, when comparing PD-FOF+ with PD- FOF−, significant differences were found for the ratios $\frac{V_{A\,L\,L,P\,95}}{V_{T\,U\,G,a\,v\,g}}$, $\frac{V_{S\,W\,B,P\,95}}{V_{T\,U\,G,a\,v\,g}}$, $\frac{V_{M\,W\,B,P\,95}}{V_{T\,U\,G,a\,v\,g}},\frac{V_{L\,W\,B,P\,50}}{V_{T\,U\,G,a\,v\,g}}$, $\frac{V_{L\,W\,B,P\,95}}{V_{T\,U\,G,a\,v\,g}}$, $\frac{\omega_{H,P\,50}}{V_{T\,U\,G,a\,v\,g}}$, and $\frac{\omega_{H,P\,95}}{\omega_{T\,U\,G,2}}$, with higher ratios in the PD-FOF+ group.

## Discussion

Most of the previous studies on this topic that have shown mobility-associated differences between PD-FOF+ and PD-FOF− have investigated their participants only in the lab. In this study, thanks to IMUs and dedicated algorithms, several mobility parameters were collected from patients with PD with and without FOF, when performing functional tests in the lab and living in their usual environment. The effect of FOF was investigated by quantifying the changes in mobility parameters between lab and daily life. The discriminative power between PD-FOF+ and PD-FOF− was shown by a logistic regression model considering each setting separately and in combination. And finally, the association between the lab and daily activity setting was studied by considering their correlation.

Regarding the effect of FOF on mobility, PD-FOF+ needed more time to perform the TUG test than the PD- FOF−, which was –at least partly- explained by the slower performance of the two turns included in this test (Table 2). This supports previous findings (Bryant et al., 2014; Haertner et al., 2018; Abou et al., 2021) and suggests that PD-FOF+ suffer from increased fear, especially during turns. This fear may be justified, e.g., through increased dysbalance or other constraints associated with FOF (Pourghayoomi et al., 2020). The larger difference between the two groups in the second turn, which also includes a stand- or walk-to-sit movement, may also argue for the different balance capacities between the groups. This argument is further supported by the slower peak angular velocity during the second turn compared with the first turn in the PD-FOF+ group.

In contrast to the evidence in the literature (Bryant et al., 2014), we did not observe a significant difference in gait speed between PD-FOF+ and PD-FOF− during the TUG test (*V*_*TUG,avg*_). As the *r*-value showed a large effect size for this parameter in both groups, we hypothesize that PD severity rather than FOF has a particular influence on this parameter. We performed a Wilcoxon rank-sum test on *V*_*TUG,avg*_ without adjusting for the aforementioned confounders, and obtained a significant difference between the PD-FOF+ and PD-FOF− (*p* = 0.021). Therefore, more evidence with a larger dataset is required to confirm this hypothesis as most of the previous studies did not adjust the statistical analysis for potential confounders.

Although none of the 5xSTS tests could sufficiently discriminate between PD-FOF+ and PD- FOF−, the fast 5xSTS test presented larger effect sizes than the preferred speed 5xSTS test (Table 2 and Figure 1). This is an argument for including the fast version rather than the preferred speed version (Goldberg et al., 2012; Staartjes and Schröder, 2018) in the assessment panel of clinical protocols. For the 5xSTS with preferred speed, the mean peak power of sit-to-stands (*P*_*5xSTS,N*_) had a medium effect size while the effect size for the total duration of the test (*T*_*5xSTS,N*_) was low (Table 2 and Figure 1). This again highlights the usefulness of an instrumented 5xSTS test with IMUs to extract biomechanical parameters beyond the conventionally measured duration of the test (Van Lummel et al., 2016). Nevertheless, the IMU-derived sit-to-stand peak power did not differentiate PD-FOF+ from PD-FOF−. Also, the sit-to-stand peak power derived from the TUG test (*P*_*TUG*_) was not significantly different between the groups. An explanation can be that the PD-FOF+ group might not have particular difficulties in performing postural transitions. However, numerous studies showed the predictive power of the 5xSTS test for future falls (Buatois et al., 2008; Duncan et al., 2011; Doheny et al., 2013; Qiu et al., 2018). Therefore, our results, together with previous results, suggest that the 5xSTS test is associated with aspects of falls that are independent of FOF.

None of the parameters derived from the daily activity assessment could significantly differentiate PD-FOF+ from PD-FOF−. However, medium effect size values were observed for several parameters. Interestingly, the effect size for the lower preferred gait speed (*V*_*μ _1*_) was higher than the median or 95th percentile values of gait speed distribution. This shows the importance of more precise modeling of gait speed distribution, rather than assuming a simple normal distribution of the obviously complex movements that occur in the usual environment (which was done in most of the previous studies, e.g., Toosizadeh et al., 2015; Takayanagi et al., 2019; Shah et al., 2020b). Interestingly, *V*_*μ _1*_ showed a higher effect size than *V*_*μ _2*_. It should be noted that *V*_*μ _1*_ is assumed to correspond more to shorter walking bouts and *V*_*μ _2*_ represents mostly longer walking bouts that are more likely to occur outdoors (Van Ancum et al., 2019). Thus, our results regarding the higher effect size of *V*_*μ _1*_ vs. *V*_*μ _2*_ suggest that shorter walking bouts are more meaningful to describe mobility performance (limitations) of PD-FOF+, and maybe an interesting therapeutic target for future trials. It could also be speculated that PD-FOF+ have more problems than PD-FOF− during multitask-walking, as shorter walking bouts have obviously a higher probability to be associated with additional tasks, compared with long walking bouts which have a high probability for reflection, e.g., walks without relevant dual-task claim. Therefore, according to Figure 1, it is not surprising that the features that remained for the classification included more parameters from short walking bouts (*Gait*_*SWB,min*_, *Gait*_*SWB,avg*_, and *Gait*_*SWB,max*_) than from medium and long walking bouts (*Gait*_*MWB,avg*_ and *Gait*_*MWB*,*max*_).

In addition to *Gait*_*ALL,max*_, *SiSt*_*max*_ and *Turn*_*min*_ were among the daily activity parameters with the highest effect sizes. Thus, the number of various types of activities should also be considered in addition to parameters such as gait speed, sit-to-stand peak power, and turning peak angular velocity that characterizes these activities. Moreover, there was a tendency toward a lower amount of activity in PD-FOF+. The PDQ score was significantly lower in PD-FOF+, showing that the quality of life of the patients was highly affected by their FOF. This can explain the lower amount of daily activities in this group of patients.

After feature selection in section “Lab Versus Daily Activity Assessment,” several parameters from the lab and daily activity assessments remained in the selected features (Figure 1). Training three classifiers based on three sets of features, i.e., *𝔽*1, *𝔽*2, and *𝔽*3, revealed that set *𝔽*1 led to the most accurate classifier to distinguish the PD-FOF+ from the PD-FOF− group (Table 4). This selection, including features from both the lab and daily activity assessments, further supports the usefulness of including daily activity assessments in clinical practice as they have complementary information to the assessments performed in the lab (Maetzler et al., 2021). The more accurate classification of FOF with lab features (*𝔽*2), compared with daily activity features (*𝔽*3, Table 4), suggests that capacity aspects play an important role for the definition of FOF (Maetzler et al., 2021) and functional tests in the lab should always be performed for the evaluation in FOF. Still, the inclusion of environmental context and psychological factors from daily life is a valuable addition and can contribute to increased specificity.

Comparing the gait speed between the lab and daily activity assessments, significant correlations were found for PD-FOF− but not PD-FOF+ (Table 5). Interestingly, PD-FOF+ had higher gait speed values in the “capacity” area of their daily activity assessment compared with the lab. For these participants, $\frac{V_{P\,95}}{V_{T\,U\,G,a\,v\,g}}$, $\frac{V_{S\,W\,B,P\,95}}{V_{T\,U\,G,a\,v\,g}}$, $\frac{V_{M\,W\,B,P\,95}}{V_{T\,U\,G,a\,v\,g}}$, and $\frac{V_{L\,W\,B,P\,95}}{V_{T\,U\,G,a\,v\,g}}$ had values greater than 1 (Table 5). One explanation can be that PD-FOF+ might be more cautious in non-familiar environments such as the lab. Moreover, and potentially more relevant for future management strategies, they might have been less cautious in their daily life especially when it comes to fast (and therefore more dangerous) gait episodes (Salkovic et al., 2017).

Another interesting observation, in our view, was that in PD- FOF−, *V*_*TUG,avg*_ was significantly correlated with parameters during daily activity assessments that represent mostly the capacity aspects, i.e., *V*_*H,P95*_, *V*_*H,μ _2*_, *V*_*MWB,P95*_, and *V*_*LWB,P50*_. Moreover, the correlation between *V*_*TUG,avg*_ and *V*_*μ _2*_was high (ρ = 0.70). These findings firstly confirm the relevant association of lab parameters with daily activity parameters that are near the capacity area (Van Ancum et al., 2019; Warmerdam et al., 2020). These results suggest that capacity-associated values obtained during daily activities can indeed predict the capacity of a participant in the lab. Furthermore, the high association between *V*_*TUG,avg*_ and *V*_*μ _2*_ is again in favor of considering a bimodal gait speed distribution during daily activities (Atrsaei et al., 2021a).

Regarding the sit-to-stand peak power, *P*_*H,P95*_ had high correlations with *P*_*TUG*_ and *P*_*5xSTS,N*_ but not with *P*_*5xSTS,F*_ (Table 5). This indicates that the 5xSTS test with preferred speed and the sit-to-stand part of the TUG test is most representative of the sit-to-stands performed during daily activities. In fact, in the TUG test, it is more accurate to name the initial postural transition as sit-to-walk rather than sit-to-stand. Since in daily life, there is often more sitting-to-walking than sitting-to-standing, the high correlation between *P*_*TUG*_ and *P*_*H,P95*_ seems reasonable. Therefore, to have a better understanding of the sit-to-stand performance of patients during daily activities, clinicians should consider the 5xSTS test with preferred speed and the TUG sit-to-stand movement, rather than the fast 5xSTS test. The high association of sit-to-stand peak power between the lab and daily activity assessments was also observed in a study in community-dwelling older adults (Zhang et al., 2017). Nevertheless, as we demonstrated earlier, the 5xSTS test with fast speed had higher discriminative power for differentiating PD-FOF+ from PD-FOF−.

Our results are comparable to a very recent study on the impact of FOF on mobility parameters in a relatively large population of community-dwelling older adults (Wang et al., 2021). In that study, FOF led to a poorer mobility performance during both lab and daily activity assessments. Moreover, and comparable to this study, the consideration of both assessments showed the best discriminatory power between the presence and absence of FOF (lab assessment, AUC = 0.64; lab and daily activity assessment, AUC = 0.77). The strengths of our study, compared with the aforementioned study, are that we included postural transition and turning in addition to walking, and we assessed the daily activity over an average period of 12 (and not only 2) days (Wang et al., 2021).

Our study faces some limitations. First, our sample size could be small for statistical analyses. The observations and results could be supported more strongly with a larger population. This could explain why the parameters obtained during the daily activities did not differ significantly between PD-FOF+ and PD-FOF−. For instance, *V*_*μ _1*_ was at the edge of a statistically significant difference. However, it should be noted that finding participants with a specific impairment that are willing to participate in several clinical assessments, as well as 2 weeks of activity monitoring, can be challenging. While in this study, we explored the difference between participants with low and moderate FOF, the difference between participants with low and high FOF might be more evident with mobility parameters obtained during daily activities. Using other questionnaires in addition to FES-I can also be investigated. For example, participants can be asked whether their FOF restricts their activities or not (Rochat et al., 2010).

The duration of daily activities measurements could still be increased to have a more accurate estimation of the daily routines of the patients. Nevertheless, considering the current usability of IMUs, especially for older adults, it is a bit challenging to engage the participants for more than 2 weeks of measurements. It is not surprising that in the literature, a lot of studies consider only 1 week of daily activity measurements (Storm et al., 2018; Galperin et al., 2019; Van Ancum et al., 2019; Shah et al., 2020a).

Another point of limitation can be the turning assessment during daily activities. The turning algorithm considered turns with durations of 0.5–10 s and angles > 45° (El-Gohary et al., 2014). This is a broad range, and future studies should investigate whether more specific definitions for turns that are performed in daily life have higher discriminatory power. Furthermore, the employed algorithm detected turns regardless of their occurrence during walking or sedentary behavior. Although it might be rare, participants might have been in a sitting position in a moving vehicle that had similar turning to those of a human that walks and turns at the same time. Therefore, further work is required to adapt the algorithm to detect turnings that occur during locomotion.

Another point was that since some of the unitless parameters showed significant differences between the two groups (Figure 2), we were curious if adding them to the feature set *𝔽*1 will improve the classification results in Table 4. However, no improvement was observed. The reason might be that the ratio of home-derived mobility parameter divided by the same parameter obtained in the lab did not bring additional information as the information regarding both of the assessment settings was already there. Finally, to keep the data accuracy as high as possible, we excluded walking bouts < 15 s from the analysis to prevent other activities from being wrongly detected as a walking bout. However, these walking bouts contribute to a relevant portion of daily walking (Del Din et al., 2016; Shah et al., 2020c), and removing them might affect the meaningfulness of walking parameters with respect to the actual research question.

To conclude, the use of the IMU along with the dedicated algorithms allowed an unobtrusive assessment of mobility during daily activities. Although lab-based mobility parameters had generally higher discriminative power in differentiating PD-FOF+ and PD- FOF−, integrating daily activity assessments provided a more accurate classification of these patients. By comparing the same parameters from both settings, we could show for the first time that (i) considering lab and daily activity mobility parameters can lead to more accurate classification of PD-FOF+ and PD-FOF− compared with each lab and daily activity assessments alone (ii) the PD-FOF+ group performs the lab assessments with a rather cautious gait but used a rather incautious gait pattern in the usual environment; (iii) the sit-to-stand peak power of the 5xSTS test with preferred speed and of the TUG was more closely associated with sit-to-stand movement in daily life, than was the same parameter obtained from the fast 5xSTS, and (iv) the 5xSTS test with fast speed mostly measured the capacity aspects of daily activities. These results provide further insight into the daily life behavior of PD patients with FOF, can stimulate prevention and treatment strategies, and can serve as a template for further studies using these novel techniques and assessment strategies.

## Data Availability Statement

The datasets generated and/or analyzed during this study are not publicly available but might be accessible from the corresponding author on reasonable request, upon joint approval from AA, CH, WM, and KA.

## Ethics Statement

The studies involving human participants were reviewed and approved by Medical Faculty of the University of Tübingen (protocol no. 686/2013BO1). The patients/participants provided their written informed consent to participate in this study.

## Author Contributions

AA designed the technical research and scientific question, extracted and analyzed the mobility parameters from clinical and home-based measurements, and wrote the manuscript. CH, ME, SS, DB, and IL-S performed the clinical measurements and extracted and analyzed the clinical questionnaires. AA, WM, DB, and KA discussed the results. WM, DB, and KA supervised and led the study. All the authors read, revised, and approved the final manuscript.

## Conflict of Interest

The authors declare that the research was conducted in the absence of any commercial or financial relationships that could be construed as a potential conflict of interest.

## Publisher’s Note

All claims expressed in this article are solely those of the authors and do not necessarily represent those of their affiliated organizations, or those of the publisher, the editors and the reviewers. Any product that may be evaluated in this article, or claim that may be made by its manufacturer, is not guaranteed or endorsed by the publisher.
